# Left ventricular subclinical myocardial dysfunction in uncomplicated type 2 diabetes mellitus is associated with impaired myocardial perfusion: a contrast-enhanced cardiovascular magnetic resonance study

**DOI:** 10.1186/s12933-018-0782-0

**Published:** 2018-10-30

**Authors:** Xi Liu, Zhi-gang Yang, Yue Gao, Lin-jun Xie, Li Jiang, Bi-yue Hu, Kai-yue Diao, Ke Shi, Hua-yan Xu, Meng-ting Shen, Yan Ren, Ying-kun Guo

**Affiliations:** 1 Department of Radiology, West China Hospital, Sichuan University, 37# Guo Xue Xiang, Chengdu, Sichuan 610041 China; 2 Department of Radiology, Key Laboratory of Obstetric & Gynecologic and Pediatric Diseases and Birth Defects of Ministry of Education, National Key Laboratory of Biotherapy, West China Second University Hospital, Sichuan University, 20# South Renmin Road, Chengdu, Sichuan 610041 China; 3 Department of Endocrinology and Metabolism, West China Hospital, Sichuan University, 37# Guo Xue Xiang, Chengdu, Sichuan 610041 China

**Keywords:** Type 2 diabetes mellitus, Cardiac magnetic resonance, Subclinical myocardial dysfunction, Myocardial perfusion

## Abstract

**Background:**

Early detection of subclinical myocardial dysfunction in patients with diabetes mellitus (DM) is essential for recommending therapeutic interventions that can prevent or reverse heart failure, thereby improving the prognosis in such patients. This study aims to quantitatively evaluate left ventricular (LV) myocardial deformation and perfusion using cardiovascular magnetic resonance (CMR) imaging in patients with type 2 diabetes mellitus (T2DM), and to investigate the association between LV subclinical myocardial dysfunction and coronary microvascular perfusion.

**Methods:**

We recruited 71 T2DM patients and 30 healthy individuals as controls who underwent CMR examination. The T2DM patients were subdivided into two groups, namely the newly diagnosed DM group (*n* = 31, patients with diabetes for ≤ 5 years) and longer-term DM group (*n* = 40, patients with diabetes > 5 years). LV deformation parameters, including global peak strain (PS), peak systolic strain rate, and peak diastolic strain rate (PSDR), and myocardial perfusion parameters such as upslope, time to maximum signal intensity (TTM), and max signal intensity (Max SI, were measured and compared among the three groups. Pearson’s correlation was used to evaluate the correlation between LV deformation and perfusion parameters.

**Results:**

Pooled data from T2DM patients showed a decrease in global longitudinal, circumferential, and radial PDSR compared to healthy individuals, apart from lower upslope. In addition, increased TTM and reduced Max SI were found in the longer-term diabetics compared to the normal subjects (*p* < 0.017 for all). Multivariable linear regression analysis showed that T2DM was independently associated with statistically significant CMR parameters, except for TTM (*β *= 0.137, *p* = 0.195). Further, longitudinal PDSR was significantly associated with upslope (*r* = − 0.346, *p* = 0.003) and TTM (*r* = 0.515, *p* < 0.001).

**Conclusions:**

Our results imply that a contrast-enhanced 3.0T CMR can detect subclinical myocardial dysfunction and impaired myocardial microvascular perfusion in the early stages of T2DM, and that the myocardial dysfunction is associated with impaired coronary microvascular perfusion.

**Electronic supplementary material:**

The online version of this article (10.1186/s12933-018-0782-0) contains supplementary material, which is available to authorized users.

## Background

Diabetes mellitus (DM), characterized by hyperglycemia, is one of the most common metabolic diseases worldwide with continuously increasing prevalence [1, 2]. A major cause of increased mortality in patients with DM is diabetic cardiomyopathy (DCM) [3, 4], which is defined as myocardial dysfunction that is independent of coronary artery disease (CAD) and hypertension and can lead to heart failure [4, 5]. Once heart failure is established, it means worse clinical outcomes in patients with diabetes [6]. The pathogenesis of DCM is complex and multifactorial, and several causative mechanisms, including metabolic effects on the myocyte, microangiopathy, and autonomic nervous dysfunction, have been postulated [5, 7, 8].

However, a substantial proportion of DM patients who do not yet satisfy DCM criteria may be at risk for progressing to DCM or heart failure. A previous research have found that impaired global longitudinal strain was associated with cardiovascular events in T2DM patients [9]. Therefore, early detection of myocardial dysfunction in patients with DM is essential for recommending therapeutic interventions that can prevent or reverse heart failure, as the severity of cardiac disease is a key indicator that determines prognosis. Even without clinically manifested heart disease, patients with type 2 diabetes mellitus (T2DM) have subtle changes in cardiac function, including left ventricular (LV) myocardial diastolic dysfunction and impaired myocardial perfusion [10–13]. With time, these subtle changes can progress to impaired systolic function or even clinical heart failure [14].

Cardiac magnetic resonance (CMR) imaging can provide a detailed and non-invasive picture of the myocardium, allowing simultaneous assessment of cardiac structure and function, and myocardial perfusion, in a single examination. Recently, feature tracking CMR (FT-CMR) imaging has emerged as a more sensitive technology for measuring myocardial deformation as an indicator of subclinical myocardial dysfunction [15]. There are numerous studies on FT-CMR imaging of T2DM that focus mainly on left ventricle deformation, with few studies investigating the left atrium [16]. Besides, first-pass myocardial perfusion MR can be used to non-invasively monitor myocardial microvascular dysfunction. Therefore, this study aims to quantitatively evaluate LV myocardial deformation and perfusion using CMR imaging in patients with T2DM and normal subjects, and to investigate the association between LV subclinical myocardial dysfunction and coronary microvascular perfusion.

## Methods and materials

### Study population

The study complied with the mandate of the Declaration of Helsinki and was approved by the Institutional Review Board of our hospital with written informed consent obtained from all study participants. From April 2016 to November 2017, we prospectively recruited 78 patients with T2DM from outpatients attending the Department of Endocrinology at our institution. T2DM was diagnosed according to the current American Diabetes Association guidelines [17]. The main exclusion criteria were clinical evidence of CAD, cardiomyopathy, myocardial infarction, or valvular heart disease (confirmed by echocardiography, electrocardiogram, or coronary computed tomographic angiography), a history of chest pain, uncontrolled hypertension (systolic blood pressure > 160 mmHg), severe renal failure (estimated glomerular filtration rate, eGFR < 30 ml/min), or contraindications to MR imaging. For comparison, 31 age- and sex-matched healthy volunteers with no history of cardiac disease or diabetes mellitus were recruited from the local community. All apparently healthy individuals who were to be recruited as controls underwent laboratory measurements before enrollment, and the exclusion criteria were impaired fasting glucose (fasting glucose > 6.1 mmol/l), dyslipidemia, or hypertension (blood pressure > 140/90 mmHg).

### Anthropometric measurements and laboratory analysis

The height and weight of all participants were recorded and BMI was calculated as weight (kg) divided by the square of height (m). The duration of diabetes was recorded as reported by the patient. Blood pressure was recorded as an average of three measurements in the right arm in a sitting position that were obtained after a 10 min resting period. Before CMR imaging, fasting (8 h) blood samples were obtained from patients and controls for standard laboratory analysis, including plasma glucose, glycosylated hemoglobin, and blood lipids, according to standard procedures of the central clinical laboratory in our hospital.

### CMR protocol

CMR imaging was performed with patients in the supine position and on a 3.0-T whole-body scanner (Trio Tim; Siemens Medical Solutions, Erlangen, Germany) with a dedicated two-element cardiac-phased array coil. A standard ECG-triggering device was also simultaneously used, and data was acquired during the breath-holding period. After the cardiac axes were determined using localizers, a balanced steady-state free-precession (bSSFP) sequence (TR/TE 39.34/1.22 ms, flip angle 38°, slice thickness 8 mm, field of view 360 × 300 mm, matrix size 256 × 166) was used to obtain cine images of the long-axis and the short-axis views and to achieve complete LV coverage from the mitral valve to the apex. For perfusion imaging, a contrast dose of 0.2 ml/kg gadobenate dimeglumine (MultiHance; Bracco, Milan, Italy) was administered using an automated injector (Stellant, MEDRAD, Indianola, PA, USA) at a flow rate of 2.5–3.0 ml/s, followed by a 20 ml saline flush at a rate of 3.0 ml/s. Rest perfusion images were acquired concurrently with intravenous contrast agents in three standard short-axis slices (apical, middle, and basal) and in one 4-chamber view slice using an inversion-recovery echo-planar imaging sequence (TR/TE 163.00/0.98 ms, flip angle 10°, slice thickness 8 mm, field of view 360 × 270 mm, matrix size 256 × 192). To exclude myocardial infarction, late gadolinium enhancement (LGE) imaging (Additional file 1) was obtained at an average of 10–15 min after contrast injection by using a segmented-turbo-FLASH–phase-sensitive inversion recovery (PSIR) sequence (TR/TE = 750.00/1.18 ms; flip angle = 40°; slice thickness = 8 mm; field of view = 400 × 270 mm^2^; matrix size = 184 × 256).

### CMR image analysis

**Fig. 1:**
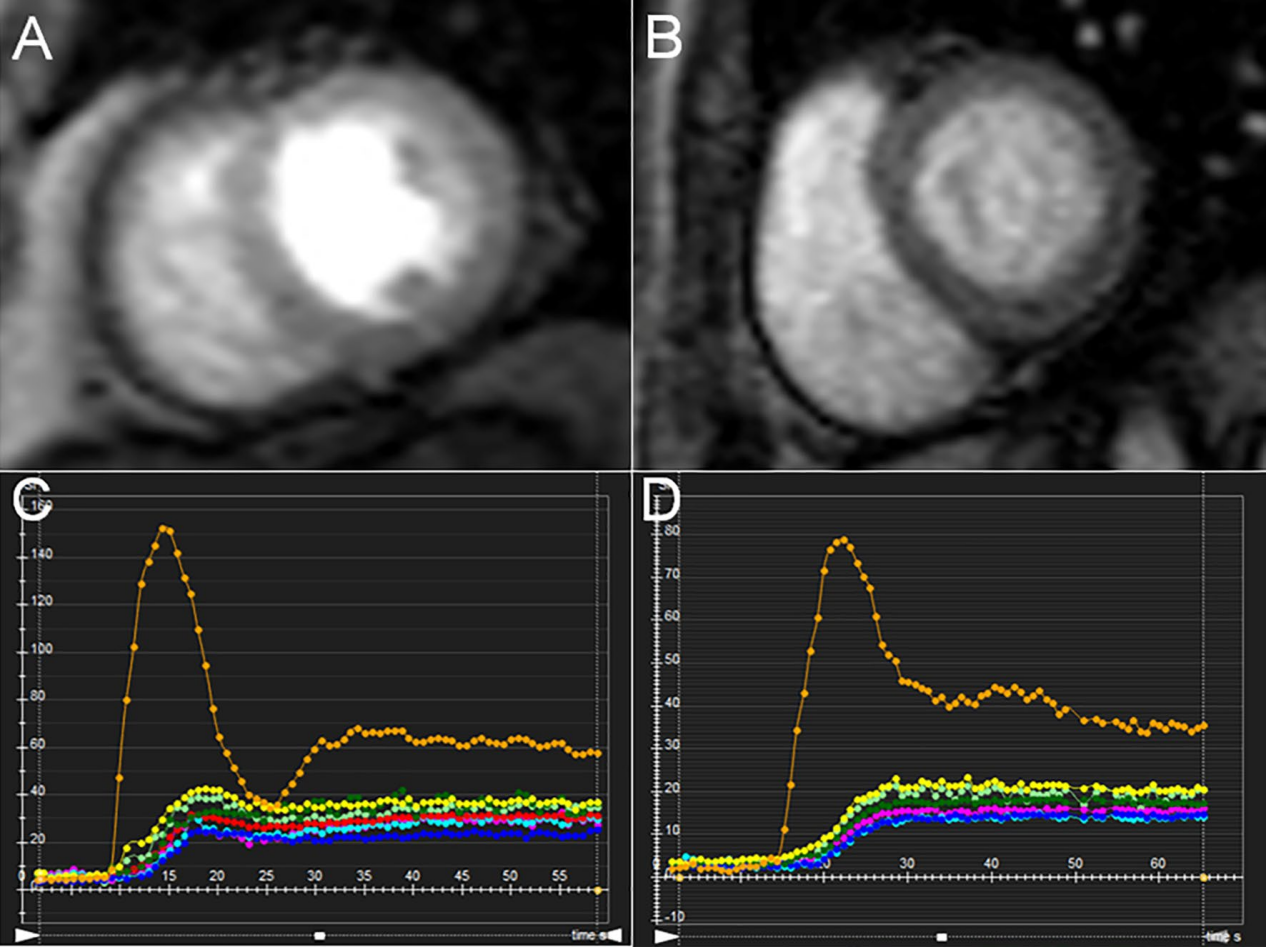
Representative first-pass myocardial perfusion MR images and signal intensity-time curves in normal controls (**A**, **C**) and patients with T2DM (**B**, **D**)

All MRI data were uploaded to commercially software (cmr42 version 5.9.1, Circle Cardiovascular Imaging Inc., Calgary, Canada), the measurements were analyzed by a investigator who was blinded to the status (DM vs control, newly diagnosed vs. longer-term DM) of the subjects. Left ventricular function parameters, namely, LV end-diastolic volume (EDV), end-systolic volume (ESV), stroke volume (SV), and ejection fraction (EF) were calculated by manually tracing the endocardial and epicardial contours in serial short-axis slices at the end-diastolic and end-systolic phases. For analysis of LV myocardial strain, long-axis 2-chamber, 4-chamber, and short-axis slices were loaded into the 3-dimensional (3D) tissue tracking module [18]. The endocardial and epicardial contours were delineated manually per slice at end-diastole phase (reference phase) in all series, while carefully excluding the papillary muscles and moderator bands. LV global myocardial strain parameters were acquired automatically, including radial, circumferential, and longitudinal peak strain (PS), peak systolic strain rate (PSSR), and peak diastolic strain rate (PDSR). For analyzing LV myocardial perfusion, endocardial and epicardial contours were delineated manually in the first-pass perfusion images of the basal, mid, and apical short-axis slices, along with a region of interest drawn in the LV blood pool. Conforming to AHA standard segmentation recommendations, a 16-segment mode (Bull’s eye plot) was constructed, which included six basal segments, six middle segments, and four apical segments [19]. Subsequently, a myocardial signal intensity-time curve was generated (Fig. 1), and LV segmental perfusion parameters such as upslope, time to maximum signal intensity (TTM), and max signal intensity (Max SI) were obtained automatically. For each subject, all LV global perfusion parameters were calculated using average regional values for the 16 myocardial segments.

### Variability analysis

To determine intraobserver variability, LV global deformation and perfusion parameters in 30 random cases that included 22 T2DM patients and 8 normal controls were measured twice in 2-week intervals by a radiologist. Then, a second investigator, who was blinded to the first investigator’s results, reanalyzed the measurements. Finally, the interobserver variability was assessed on the basis of the two investigators’ results.

### Statistical analysis

All statistical analyses were performed with SPSS statistics for Windows (Version 17.0; SPSS Institute, Inc., Chicago, IL, USA). All data were evaluated for normality using the Kolmogorov–Smirnov test and are presented as mean ± standard deviation. Homogeneity of variance was evaluated using the Levene’s test. One-way analysis of variance (one-way ANOVA) was used to compare LV function, deformation, and perfusion parameters among controls, newly diagnosed diabetics, and longer-term diabetics, while, Least-Significant-Difference was used to analyze the difference between pooled data of the diabetes and normal groups, and the Student–Newman–Keuls or Least—Significant Difference test was used to evaluate the difference within the diabetes groups when the p-value of one-way ANOVA was less than 0.05. The Kruskal–Wallis rank test was used to analyze parameters that did not conform to normality or show homogeneity of variance. Binary variables were analyzed using the cross tabs Chi square test. Pearson’s correlation was used to examine the correlation between LV strain and perfusion parameters. The intraclass correlation coefficient (ICC) was used to evaluate both inter- and intra-observer variability.

Univariable analyses were performed to demonstrate the relationship between strain and perfusion or statistically significant CMR parameters and the presence of DM as well as other risk factors. Variables with a probability value of 0.1 in the univariable analyses were then included in a stepwise multivariable analysis based on a linear regression model. A *p*-value of < 0.05 was considered statistically significant.

## Results

### Participant characteristics

We recruited 109 subjects (78 patients and 31 controls) for the study and acquired CMR imaging data for all subjects. However, data from five subjects had to be excluded because of poor image quality (e.g., severe motion artifacts in four T2DM patients and one control) and from three patients because the inferior quality of the perfusion data sets prevented quantitative analysis. Thus, the final study cohort comprised 71 T2DM patients and 30 healthy controls (proportion of males 56.7% vs. 57.7%, *p* = 0.92; mean age 53.99 ± 11.15 vs. 53.23 ± 8.59 years, *p* = 0.74). Based on the duration of diabetes [7, 20], all T2DM patients were categorized as either newly diagnosed DM (duration of diabetes ≤ 5 years, *n* = 31) or longer-term DM (duration of diabetes > 5 years, *n* = 41). The baseline characteristics of the T2DM patients and healthy volunteers are presented in Table 1. Compared to the newly diagnosed group, characteristics such as proportion of males, BMI, and systolic blood pressure were statistically higher in the longer-term DM cohort, while systolic blood pressure was significantly higher in the newly diagnosed DM group compared to the control group (*p* < 0.05). Fasting plasma glucose, glycated hemoglobin, and triglyceride levels were higher in patients with T2DM compared to healthy subjects but were not different between the two subgroups of T2DM patients. As expected, diabetic patients had lower high-density lipoprotein (HDL) and lower low-density lipoprotein compared to healthy controls. Total cholesterol was also significantly lower in newly diagnosed DM patients than in control subjects, which might be due to the fact that a significant proportion of diabetics were also on statin therapy.

**Table 1:** Baseline characteristics of the study cohort

	Normal n = 30	Newly diagnosed DM n = 31	Longer-term DM n = 40
Age (years)	53.23 ± 8.59	50.74 ± 11.92	56.50 ± 9.96
Male gender, n (%)	17 (56.7%)	12 (38.7%)	29 (72.5%)^§^
BMI (kg/m^2^)	22.44 ± 1.61	23.08 ± 3.19	24.53 ± 2.82*^§^
Diabetes duration (years)		2.24 ± 1.76*	11.45 ± 3.52*^§^
Systolic blood pressure (mmHg)	118.47 ± 7.86	125.58 ± 9.54*	131.13 ± 13.03*^§^
Diastolic blood pressure (mmHg)	78.37 ± 6.87	80.58 ± 9.41	80.53 ± 9.04
Rest heart rate (bpm)	79.07 ± 8.35	76.61 ± 11.83	74.00 ± 10.52
Fasting plasma glucose (mmol/l)	5.11 ± 0.32	7.57 ± 2.01*	7.92 ± 1.83*
Glycated haemoglobin (%)	5.49 ± 0.48	7.22 ± 1.44*	7.81 ± 1.80*
Plasma triglycerides (mmol/l)	1.16 ± 0.29	1.57 ± 0.54*	1.63 ± 0.83*
Total cholesterol (mmol/l)	4.36 ± 0.60	3.94 ± 0.88*	4.44 ± 1.16^§^
HDL (mmol/l)	1.45 ± 0.26	1.23 ± 0.29*	1.27 ± 0.50*
LDL (mmol/l)	2.97 ± 0.29	2.22 ± 0.69*	2.53 ± 0.83*

Data given as the mean ± SD

Newly diagnosed DM, patients with duration of diabetes < 5 years; Longer-term DM, patients with duration of diabetes > 5 years; BMI, body mass index; HDL, high-density lipoprotein cholesterol; LDL, low-density lipoprotein cholesterol

* p < 0.05 versus normal group (LSD)

^§^ p < 0.05 versus newly diagnosed DM patients (Student–Newman–Keuls)

### CMR imaging results

**Fig. 2:**
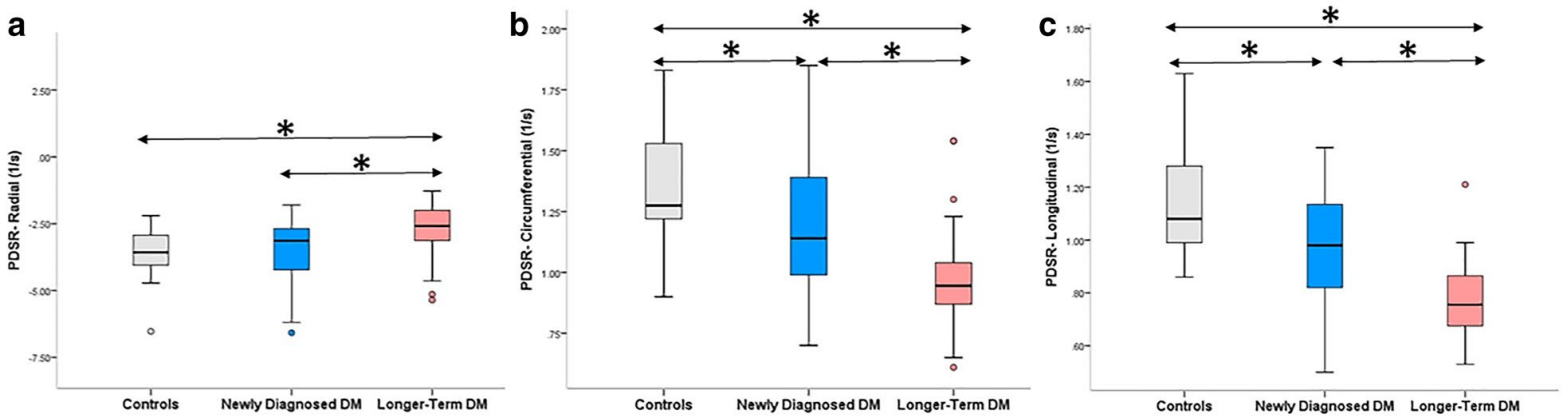
Differences in global radial (**a**), circumferential (**b**), and longitudinal PDSR (**c**) among patients with newly diagnosed DM, longer-term DM, and normal subjects. The dots indicate values outside the interquartile range, *p < 0.017

CMR imaging results for LV volume and function are summarized in Table 2. There were no significant differences in LVEDVI (LVEDV index), LVESVI (LVESV index) and LVEF among control subjects, newly diagnosed diabetics, and longer-term diabetics. However, global longitudinal PS was significantly lower in longer-term DM compared to normal subjects (*p* < 0.017). Within the T2DM patient subgroups, the global longitudinal PSSR was significantly lower in the longer-term DM group than the newly diagnosed group (*p* < 0.017). Additionally, T2DM patients showed lower global radial, circumferential, and longitudinal PDSR compared to control subjects (all *p* < 0.017), except for radial PDSR in the newly diagnosed diabetics (*p* > 0.017). Within the two subgroups of T2DM patients, the global radial, circumferential, and longitudinal PDSR were also significantly lower in the longer-term group compared to the newly diagnosed group (*p* < 0.017 for all; Fig. 2).

**Table 2:** CMR findings between normal individuals, newly diagnosed and longer-term DM patients

	Normal n = 30	Newly diagnosed DM n = 31	Longer-term DM n = 40
LVEDVI, ml/m^2^	76.95 ± 12.31	74.68 ± 11.55	73.31 ± 10.38
LVESVI, ml/m^2^	28.70 ± 5.23	30.09 ± 10.22	30.41 ± 9.36
LVSVI, ml/m^2^	48.24 ± 8.06	44.59 ± 8.26	41.54 ± 9.20*
LVEF, %	62.70 ± 3.39	60.05 ± 8.45	58.86 ± 8.93
Upslope	2.66 ± 0.56	2.12 ± 0.90*	1.81 ± 0.87*
TTM (s)	31.45 ± 5.45	33.91 ± 11.73	37.85 ± 11.03*
Max SI	22.95 ± 4.29	19.84 ± 5.11	17.98 ± 7.91*^§^
PS (%)			
Radial	43.33 ± 8.50	44.98 ± 9.11	41.95 ± 10.85
Circumferential	− 18.97 ± 1.38	− 19.27 ± 2.76	− 18.12 ± 2.80
Longitudinal	− 16.51 ± 2.16	− 16.12 ± 2.80	− 14.72 ± 2.48*
PSSR (1/s)			
Radial	2.49 ± 0.51	2.74 ± 0.73	2.46 ± 0.97
Circumferential	− 0.99 ± 0.16	− 0.98 ± 0.19	− 0.92 ± 0.22
Longitudinal	− 0.85 ± 0.18	− 0.88 ± 0.20	− 0.75 ± 0.15^§^
PDSR (1/s)			
Radial	− 3.47 ± 0.89	− 3.56 ± 1.31	− 2.60 ± 1.41*^§^
Circumferential	1.35 ± 0.25	1.20 ± 0.28*	0.97 ± 0.20*^§^
Longitudinal	1.14 ± 0.20	0.97 ± 0.21*	0.77 ± 0.14*^§^

Data given as the mean ± SD

LV, left ventricular; EDV, end diastolic volume; ESV, end systolic volume; SV, stroke volume; I, indexed to BSA; EF, ejection fraction; Time Max, time to maximum signal intensity; Max SI, max signal intensity; PS, Peak Strain; PSSR, Peak Systolic Strain Rate; PDR, Peak Diastolic Strain Rate

* p < 0.017 versus normal group (LSD)

^§^ p < 0.017 versus newly diagnosed DM patients (LSD)

**Fig. 3:**
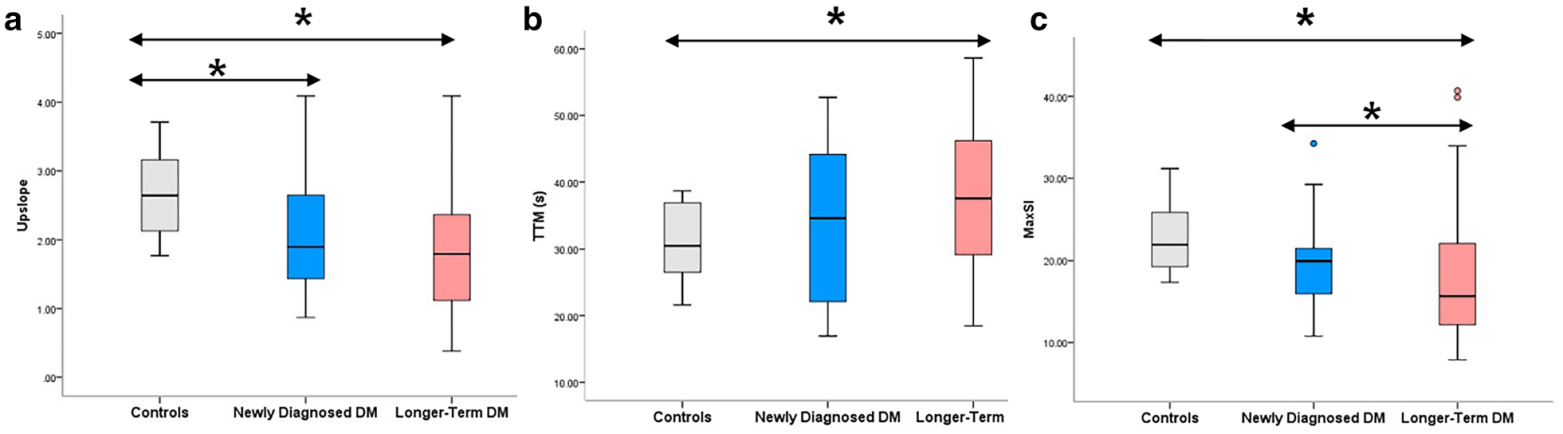
Differences in upslope (**a**), TTM (**b**), and Max SI (**c**) between patients with newly diagnosed DM, longer-term DM, and normal subjects. The dots indicate values outside the interquartile range, *p < 0.017

Data on global first-pass perfusion parameters for all subjects are presented in Table 2. Compared to the normal subjects, the upslope in the T2DM patients was significantly reduced (*p* < 0.017). Next, the TTM was higher in the longer-term DM group than the normal subjects (37.85 ± 11.03 vs. 31.45 ± 5.45, *p* < 0.017). Additionally, Max SI was significantly reduced in the longer-term diabetics compared to the newly diagnosed diabetics and the normal subjects (*p* < 0.017 for all; Fig. 3).

Multivariable linear regression analysis revealed that considering covariates (systolic blood pressure, age, sex, and BMI), T2DM was independently associated with longitudinal PS (*β *= 0.263, *p* = 0.007, model *R*^2^ = 0.11), the PSSR (*β *= 0.243, *p* = 0.014, model *R*^2^ = 0.05), and all three directions (radial, circumferential, and longitudinal) of the PSSR (*β *= 0.255, *p* = 0.008, model *R*^2^ = 0.05; *β *= 0.0.560, *p* < 0.001, model *R*^2^ = 0.31; and *β *= 0.657, *p* < 0.001, model *R*^2^ = 0.43, respectively). T2DM was also found to be independently associated with first-pass perfusion parameters (upslope: *β *= − 0.399, *p* < 0.001, model *R*^2^ = 0.15; Max SI: *β *= –0.316, *p* < 0.001, model *R*^2^ = 0.09), except for TTM (*β *= 0.137, *p* = 0.195, model *R*^2^ = 0.11).

### Association between LV deformation and first perfusion in type 2 diabetes mellitus patients

**Fig. 4:**
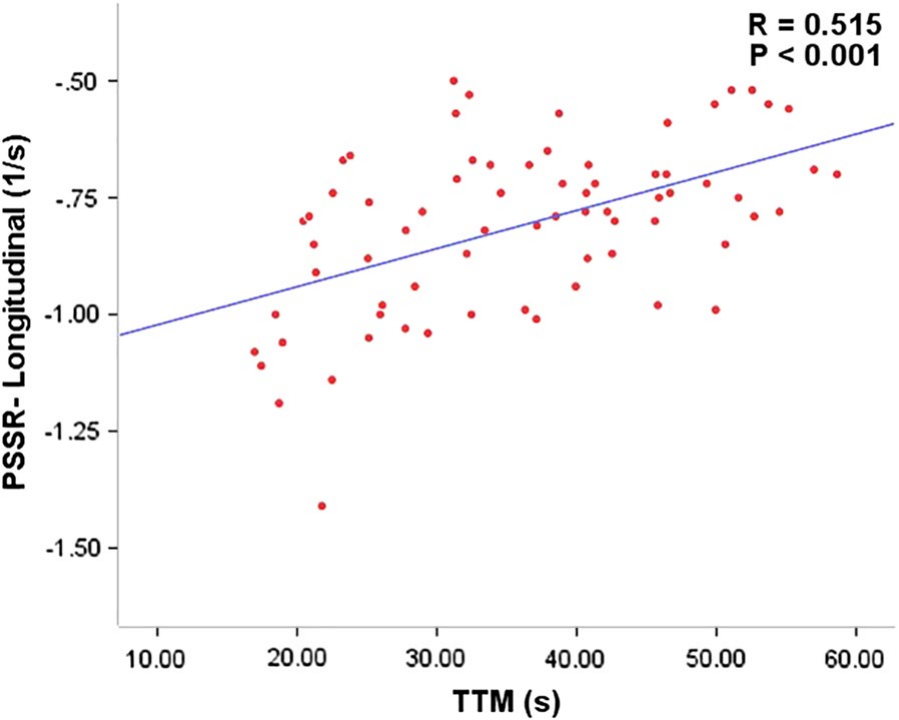
Relationship between longitudinal PSSR and TTM

As shown in Table 3, there was a weak correlation between decreased LVEF and increased TTM in the T2DM patients (r = − 0.306, *p* = 0.01). LV global radial PS and PSSR, and circumferential PDSR were inversely correlated to TTM (*r* = − 0.334, *p* = 0.004; *r* = − 0.342, *p* = 0.004; *r* = − 0.367, *p* = 0.002, respectively) in patients with T2DM. In contrast, longitudinal PS, circumferential PSSR, and radial PDSR were positively associated with TTM (*r* = 0.378, *p* = 0.001; *r* = 0.355, *p* = 0.002; *r* = 0.389, *p* = 0.001, respectively). Further, longitudinal PSSR was significantly associated with upslope (*r* = − 0.346, *p* = 0.003) and TTM (*r* = 0.515, *p* < 0.001; Fig. 4).

**Table 3:** Correlation analysis of LV deformation parameters with first-perfusion parameters in DM patients

	Upslope	p value	TTM (s)	p value	Max SI	p value
	r		r		r	
LVEF	0.212	0.076	− 0.306	0.010	0.127	0.290
PS (%)						
Radial	0.171	0.154	− 0.334	0.004	0.021	0.863
Circumferential	− 0.194	0.106	0.300	0.011	− 0.065	0.588
Longitudinal	− 0.165	0.168	0.378	0.001	0.018	0.882
PSSR (1/s)						
Radial	0.100	0.404	− 0.342	0.004	− 0.054	0.657
Circumferential	− 0.187	0.119	0.355	0.002	− 0.008	0.947
Longitudinal	− 0.346	0.003	0.515	0.000	− 0.159	0.186
PDSR (1/s)						
Radial	− 0.069	0.566	0.389	0.001	0.151	0.207
Circumferential	0.231	0.053	− 0.367	0.002	0.069	0.569
Longitudinal	0.107	0.375	− 0.255	0.032	0.043	0.724

Multivariable analysis linear regression demonstrated that TTM was independently associated with longitudinal PS (*β* = 0.378, *p* = 0.001, model *R*^2^ = 0.13) and PSSR (*β* = 0.466, *p* < 0.001, model *R*^2^ = 0.33). In addition, the upslope was independently associated with the longitudinal PSSR (*β* = − 0.291; *p* = 0.009, model *R*^2^ = 0.19; Table 4).

**Table 4:** Univariable and multivariable linear regression analysis of all patients

	Longitudinal PS		Longitudinal PSSR		Longitudinal PDSR	
	Univariable β	Multivariable β	Univariable β	Multivariable β	Univariable β	Multivariable β
Upslope/TTM/Max SI	− 0.165/0.378*/0.018	− /0.378^§^/−	− 0.346*/0.515*/− 0.159	− 0.291^§^/0.466^§^/−	0.107/− 0.255*/0.043	− /− 0.171/−
Diabetes duration	0.258*	0.258^§^/0.199/0.258^§^	0.366*	0.316^§^/0.286^§^/0.366^§^	− 0.517*	− 0.517^§^/− 0.517^§^/− 0.517^§^
Systolic blood pressure	0.133	–	0.234*	0.114/0.004/0.157	− 0.168	–
Age	− 0.106	–	0.205*	0.105/0.105/0.119	− 0.086	–
Sex	− 0.221*	− 0.151/− 0.167/− 0.151	− 0.130	–	0.272*	0.109/0.109/0.109
BMI	− 0.009	–	− 0.085	–	− 0.121	–

Factors with p < 0.1 in the univariable analysis were included in the multivariable analysis

* p < 0.1

^§^ p < 0.05

### Inter- and intra-observer variability

Table 5 summarizes the inter- and intra-observer variability for feature tracking and first-pass perfusion analysis. The ICCs for intra- and interobserver variability were 0.828–0.952 and 0.777–0.925, respectively, in feature tracking and 0.979–0.987 and 0.977–0.983, respectively, in first-pass perfusion, suggesting that both techniques are in agreement.

**Table 5:** Inter- and intra-observer variability of first-perfusion and tissue tracking

	Intra-observer (n = 30)	95% CI	Inter-observer (n = 30)	95% CI
	ICC		ICC	
Upslope	0.979	0.957–0.990	0.977	0.952–0.989
TTM (s)	0.987	0.973–0.994	0.983	0.964–0.992
Max SI	0.983	0.965–0.992	0.982	0.962–0.992
PS (%)				
Radial	0.952	0.899–0.977	0.920	0.833–0.962
Circumferential	0.934	0.862–0.968	0.915	0.823–0.960
Longitudinal	0.864	0.717–0.935	0.849	0.316–0.948
PSSR (1/s)				
Radial	0.938	0.871–0.970	0.925	0.843–0.964
Circumferential	0.866	0.720–0.936	0.847	0.596–0.934
Longitudinal	0.828	0.641–0.918	0.777	0.285–0.913
PDSR (1/s)				
Radial	0.936	0.867–0.969	0.905	0.799–0.955
Circumferential	0.881	0.751–0.943	0.871	0.732–0.938
Longitudinal	0.883	0.756–0.944	0.824	0.633–0.916

ICC, intraclass correlation coefficient

All p < 0.001

## Discussion

In the coming decades, the rising prevalence of diabetes mellitus is predicted to significantly increase mortality [2]. Therefore, early identification of subclinical myocardial dysfunction in patients with T2DM is essential for recommending targeted therapeutic strategies to reverse or alleviate this process and for predicting prognosis, both of which can provide long-term benefits against morbidity and mortality in these patients. Further, the pathogenesis of DCM appears to have several causative mechanisms and the exact underlying processes remain unidentified [5, 7, 8]. A previous study has demonstrated greater risk of heart failure in diabetic patients with retinopathy, suggesting a possible microvascular etiology of myocardial dysfunction in DCM [21]. Moreover, it has been shown that alterations in myocardial and vascular integrity, including capillary basement membrane thickening and endothelial swelling and/or degeneration, are initiated during the pre-diabetic stage [22, 23]. Therefore, we hypothesized that impaired coronary microvasculature might lead to myocardial dysfunction in DM, which raises the possibility of future implementation of therapeutic interventions with increasing microvascular function to improve the prognosis of this large patient group.

Previous studies have found that speckle tracking echocardiography (STE) can measure subclinical myocardial dysfunction in patients with T2DM at the preclinical stage; importantly, these subtle alterations are considered to be early signs of DCM [24–26]. Moreover, some studies have reported reduced coronary blood flow reserve in T2DM patients without apparent CAD, which may be another etiological factor of DCM [10, 12, 22, 23]. However, echocardiography is limited by the acoustic window, low spatial resolution, and observer dependency. Recently, feature tracking CMR (FT-CMR) imaging has emerged as a new technology that is based on routine cine CMR images and can quantitatively measure myocardial systolic and diastolic function. As it does not need specific sequences, it is possible to include this in routine use [27, 28]. Besides, recent developments in first-pass myocardial perfusion CMR have enabled the non-invasive use of this modality to evaluate coronary microvascular function with high reliability and reproducibility [29, 30]. Further, contrast-enhanced CMR (CE-CMR) imaging permits the simultaneous evaluation of both myocardial perfusion and function in a single examination. Thus, in this study, we used a CE-CMR protocol that included the feature tracking technique and the first-pass perfusion technique in subjects with newly diagnosed DM, longer-term DM, and healthy individuals to assess relative changes in myocardial perfusion and function.

Our results show that there is no significant difference in LVEF between the healthy individuals and uncomplicated T2DM patients, regardless of the duration of T2DM. However, a previous study used Doppler echocardiography to demonstrate LV diastolic dysfunction in asymptomatic type 2 diabetic patients with good glycemic control and normal EF [31], indicating that conventional cardiac function assessments are not sufficiently sensitive to early myocardial dysfunction in T2DM patients.

Next, we find reduced longitudinal PS but preserved circumferential and radial PS in longer-term diabetics compared to normal subjects. This is probably because the longitudinal myocardial fibers are predominantly located in the sub-endocardium and this wall layer is most susceptible to microvascular ischemia; thus change can lead to a reduction in longitudinal LV mechanics in the early stages of T2DM [32, 33]. Further, T2DM patients show a decrease in global longitudinal, circumferential, and radial PDSR compared to normal subjects, confirming that PDSR is more sensitive to subclinical myocardial dysfunction than either PS or PSSR in T2DM. However, a previous study using STE has shown that LV radial and circumferential diastolic functions are preserved in uncomplicated T2DM patients [26], a finding that is contradicted in our study. Therefore, we hypothesize that compared to STE, the PDSR measured by FT-CMR is more accurate and sensitive to subclinical diastolic function. Additionally, global circumferential and longitudinal PDSR are also significantly lower in longer-term DM patients compared to newly diagnosed diabetics, suggesting that diastolic dysfunction begins in the initial stage of diabetes and intensifies as the duration of diabetes increases.

Our observations of upslope, Max SI and TTM confirm impaired coronary microcirculation in T2DM patients and suggest that myocardial microvascular dysfunction begins in the early stages of T2DM and accumulates as disease duration increases; this is consistent with previously reported pathological results from animal experiments [34, 35]. Further, these results also imply that first-pass myocardial perfusion CMR can not only detect impaired coronary microcirculation early on, but also quantitatively evaluate the degree of microcirculatory damage.

A previous study has identified an association between impaired myocardial perfusion reserve and diastolic dysfunction in patients with T2DM using echocardiography and myocardial perfusion scintigraphy [36]. In contrast, another report indicated that LV diastolic function was not correlated with myocardial perfusion reserve in T2DM patients with preserved systolic LV function [37]. Furthermore, using myocardial contrast echocardiography and strain rate imaging, Moir et al. found no significant relationship between the myocardial blood flow reserve and PSSR in T2DM patients [38]. In our study, multivariable regression analysis showed that TTM and upslope were independently associated with longitudinal PSSR, suggesting a possible mechanistic link between impaired myocardial perfusion and subclinical myocardial dysfunction in patients with T2DM. This result also supports the hypothesis that impaired coronary microcirculation may contribute to subclinical myocardial dysfunction, and highlights the need for further research on the underlying mechanisms and for interventional trials aimed at reversing myocardial microvascular dysfunction that can potentially improve myocardial function and prognosis in patients with T2DM.

## Limitations

There are several limitations to our study. First, the patient population comprised uncomplicated T2DM patients in stable condition with no other significant co-morbidities; this has reduced the applicability of our results to “real world” populations with high incidence of co-morbidities. However, we recruited such participants as this design excluded the effects of confounding factors, such as other co-morbidities that affect cardiac function, and helped better explore the influence of T2DM on the myocardium. Second, due to the likely selection bias, the values of eGFR were normal in most T2DM patients. Therefore, we did not discuss the effect of eGFR on LV deformation and myocardial perfusion. Third, not all DM patients underwent coronary computed tomographic angiography (CCTA) because of the radiation dose. However, patients suspected of CAD (by CMR, echocardiography, or electrocardiogram) underwent CCTA examination. Finally, as this was a cross-sectional study, there are inherent design limitations and our results remain to be verified by longitudinal studies in T2DM patients. We aim to accomplish this in our future research endeavors.

## Conclusions

We show that 3.0T CE-CMR can simultaneously detect subclinical myocardial dysfunction and impaired myocardial microvascular perfusion in a single examination in the early stages of T2DM, and that these changes accumulate gradually over time. Further, we confirmed the correlation between impaired microvascular perfusion and subclinical myocardial dysfunction, suggesting that impaired coronary microcirculation may lead to cardiac dysfunction; this implies that future therapeutic strategies should focus on preserving or increasing coronary microvascular perfusion to prevent myocardial dysfunction in T2DM patients.

## Additional file

**Additional file 1.** The short-axis and four-chamber long-axis LGE images showed no delayed enhancement, demonstrating that the T2DM patient (corresponding to Fig. 1) have no silent ischemia.

## Abbreviations

DM: diabetes mellitus
LV: left ventricular
CMR: cardiovascular magnetic resonance
T2DM: type 2 diabetes mellitus
PS: peak strain
PSSR: peak systolic strain rate
PSDR: peak diastolic strain rate
TTM: time to maximum signal intensity
Max SI: max signal intensity
DCM: diabetic cardiomyopathy
CAD: coronary artery disease
STE: speckle tracking echocardiography
FT-CMR: CMR feature tracking imaging
eGFR: estimated glomerular filtration rate
ADA: American Diabetes Association
bSSFP: balanced steady-state free-precession
EDV: end-diastolic volume
ESV: end-systolic volume
SV: stroke volume
EF: ejection fraction
HDL: high-density lipoprotein
LDL: lower low-density lipoprotein
CE-CMR: contrast-enhanced CMR
CCTA: coronary computed tomographic angiography
