# Supplementary material for: Left ventricular subclinical myocardial dysfunction in uncomplicated type 2 diabetes mellitus is associated with impaired myocardial perfusion: a contrast-enhanced cardiovascular magnetic resonance study
Source: Cardiovasc Diabetol. 2018 Oct 30;17:139. doi: 10.1186/s12933-018-0782-0 (PMC6206833; doi:10.1186/s12933-018-0782-0)
Supplement: Supplementary file 1 — Additional file 1. The short-axis and four-chamber long-axis LGE images showed no delayed enhancement, demonstrating that the T2DM patient (corresponding to Fig. 1) have no silent ischemia. [file 12933_2018_782_MOESM1_ESM.doc]

**Correlation analysis of eGFR with LV deformation parameters as well as first-perfusion parameters in DM patients.**

|  |  | eGFR |  |  |
| --- | --- | --- | --- | --- |
|  |  | r |  | P value |
| Upslope |  | 0.157 |  | 0.191 |
| TTM (s) |  | -0.071 |  | 0.558 |
| Max SI |  | 0.075 |  | 0.533 |
| **PS (%)** |  |  |  |  |
| Radial |  | -0.046 |  | 0.704 |
| Circumferential |  | 0.049 |  | 0.687 |
| Longitudinal |  | 0.003 |  | 0.979 |
| **PSSR (1/s)** |  |  |  |  |
| Radial |  | 0.005 |  | 0.967 |
| Circumferential |  | -0.057 |  | 0.639 |
| Longitudinal |  | -0.168 |  | 0.161 |
| **PDSR (1/s)** |  |  |  |  |
| Radial |  | -0.070 |  | 0.561 |
| Circumferential |  | 0.132 |  | 0.274 |
| Longitudinal |  | 0.204 |  | 0.088 |
